# *catena*-Poly[2-methyl­pyridinium [tungstate-di-μ-selenido-silver-di-μ-selenido] 2-methyl­pyridine monosolvate]

**DOI:** 10.1107/S1600536813028213

**Published:** 2013-10-19

**Authors:** Lu-Jun Zhou, Hua-Tian Shi, Chao Xu, Qun Chen, Qian-Feng Zhang

**Affiliations:** a Department of Applied Chemistry, School of Petrochemical Engineering, Changzhou University, Jiangsu 213164, People’s Republic of China; b Institute of Molecular Engineering and Applied Chemistry, Anhui University of Technology, Ma’anshan, Anhui 243002, People’s Republic of China

## Abstract

The title compound, {(C_6_H_8_N)[AgWSe_4_]·C_6_H_7_N}_*n*_, consists of anionic [WAgSe_4_]_*n*_ chains, 2-methyl­pyridinium cations and neutral 2-methyl­pyridine mol­ecules. The Se atoms bridge the Ag and W atoms, forming a polymeric chain extending along the *b-*axis direction. Both the Ag and W atoms are located on a twofold rotation axis and each metal atom is coordinated by four Se atoms in distorted tetra­hedral geometry. In the crystal, the 2-methyl­pyridinium cation and 2-methyl­pyridine mol­ecule are linked *via* N—H⋯N hydrogen bonding. Weak C—H⋯Se inter­actions link the organic components and polymeric anions into a three-dimensional architecture.

## Related literature

For applications of compounds with [*M*,*M*′Se_4_] anions (*M*,*M*′ = transition metals), see: Zhang *et al.* (2002 ▶, 2006 ▶). For related structures, see: Huang *et al.* (1997 ▶); Lang *et al.* (1993 ▶); Müller *et al.* (1983 ▶); Yu *et al.* (1998 ▶); Dai *et al.* (2007 ▶); Zhang *et al.* (2000 ▶).
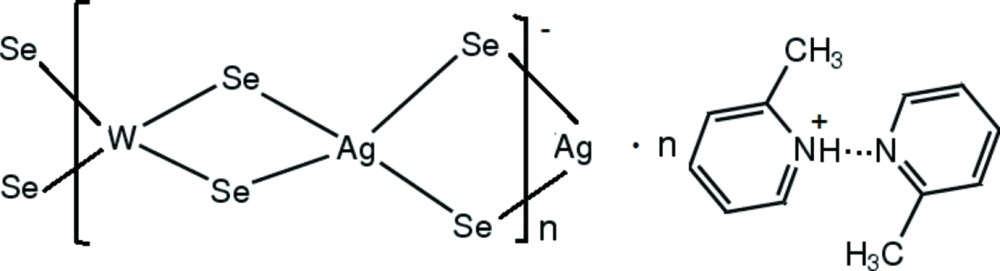


## Experimental

### 

#### Crystal data

(C_6_H_8_N)[AgWSe_4_]·C_6_H_7_N
*M* *_r_* = 794.82
Monoclinic, 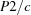

*a* = 7.859 (2) Å
*b* = 5.9448 (15) Å
*c* = 19.830 (5) Å
β = 100.962 (3)°
*V* = 909.5 (4) Å^3^
*Z* = 2
Mo *K*α radiation
μ = 15.39 mm^−1^
*T* = 296 K
0.15 × 0.12 × 0.03 mm

#### Data collection

Bruker APEXII CCD area-detector diffractometer
Absorption correction: multi-scan (*SADABS*; Bruker, 2001 ▶) *T*_min_ = 0.206, *T*_max_ = 0.655
5398 measured reflections
2051 independent reflections
1565 reflections with *I* > 2σ(*I*)
*R*_int_ = 0.041

#### Refinement

*R*[*F* ^2^ > 2σ(*F* ^2^)] = 0.034
*wR*(*F* ^2^) = 0.078
*S* = 0.97
2051 reflections
93 parameters
H-atom parameters constrained
Δρ_max_ = 0.86 e Å^−3^
Δρ_min_ = −1.06 e Å^−3^

### 

Data collection: *APEX2* (Bruker, 2007 ▶); cell refinement: *SAINT* (Bruker, 2007 ▶); data reduction: *SAINT*; program(s) used to solve structure: *SHELXTL* (Sheldrick, 2008 ▶); program(s) used to refine structure: *SHELXTL*; molecular graphics: *SHELXTL*; software used to prepare material for publication: *SHELXTL*.

## Supplementary Material

Crystal structure: contains datablock(s) I, global. DOI: 10.1107/S1600536813028213/xu5746sup1.cif

Structure factors: contains datablock(s) I. DOI: 10.1107/S1600536813028213/xu5746Isup2.hkl

Additional supplementary materials:  crystallographic information; 3D view; checkCIF report

## Figures and Tables

**Table 1:** Selected bond lengths (Å)

W1—Se1	2.3347 (9)
W1—Se2	2.3379 (9)
Ag1—Se1^i^	2.6224 (11)
Ag1—Se2	2.6210 (10)

Symmetry code: (i) 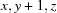.

**Table 2:** Hydrogen-bond geometry (Å, °)

*D*—H⋯*A*	*D*—H	H⋯*A*	*D*⋯*A*	*D*—H⋯*A*
N1—H1*N*⋯N1^ii^	0.86	1.93	2.786 (12)	172
C1—H1⋯Se1	0.93	2.96	3.732 (8)	141
C4—H4⋯Se1^iii^	0.93	2.90	3.832 (8)	176

Symmetry codes: (ii) 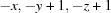; (iii) 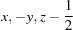.

**Fig. 1.**
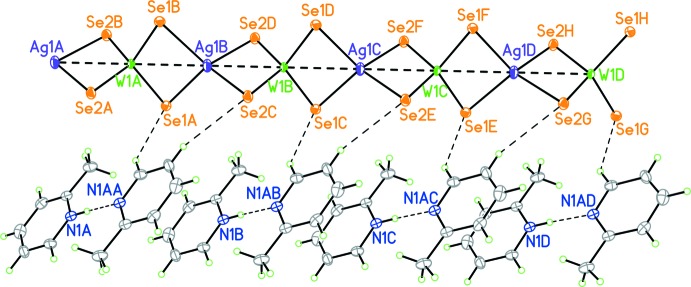
A perspective view of molecular structure of heteroselenometallic polymeric complex {[(2-MepyH)(2-Mepy)][(µ-WSe4)Ag]}n. The ellipsoids are drawn at the 50% probability level. Hydrogen bonds are shown in the dash lines.

## supplementary crystallographic information

### 1. Comment

Tetraselenometalates [MSe_4_]^2-^ (*M* =Mo, W) have been extensively used in the synthesis of heterselenometallic clusters with third-order nonlinear properties (Zhang *et al.*, 2002). Of special which argentoselenometallic clusters are of good photostability and relatively stable optical limiting effects (Zhang *et al.*, 2006). It has been found that the assembling of [MS_4_]^2-^ (*M* =Mo, W) and Ag^+^ is flexible through non-bonding interactions with complementary small molecules (or cations) and the solvent, which can assemble into polymeric heterothiometallic clusters with different configurations, such as single linear, zigzag and helical and double chains (Huang *et al.*, 1997; Lang *et al.*, 1993; Müller *et al.*, 1983; Yu *et al.*, 1998). However, it has been noted that the difficulty in the synthesis of argentoselenometallic clusters is probably due to the low solubility of Ag^+^ species that are involved in the self-assembly with the [MSe_4_]^2-^ (*M* =Mo, W) anion. It is thus understood that only two examples of structurally characterized argentoselenometallic clusters including one-dimensional linear {[Et_4_N][(µ-WSe_4_)Ag]}_n_ (Dai *et al.*, 2007) and helical {[La(Me_2_SO)_8_][(µ_3_-WSe_4_)_3_Ag_3_]}_n_ (Zhang *et al.*, 2000) have been appeared up to date. The one-dimensional chain structure of the title heteroselenometallic polymer {[(2-MepyH)(2-Mepy)][(µ-WSe_4_)Ag]}_n_ is herein described as an addition of this family.

The title heteroselenometallic complex crystallizes in the monoclinic with *P*2/*c* space group. An analogous heterothiometallic complex, {[\>a-MepyH][MoS_4_Ag]}_n_, has been reported, which crystallizes in a monoclinic *Pc* space group (Lang *et al.*, 1993). The structure determination shows that the title heteroselenometallic complex consists of [(2-MepyH)(2-Mepy)]_+_ (py = pyridine) cations with the N—H···N hydrogen bonds and polymeric linear chain of [(µ-WSe_4_)Ag]^-^ anions, as shown in Fig. 1. The anion chain is composed of extended rhombic networks of co-planar [Ag(µ-Se_2_)W] units and the neighbouring rhombi in the chain are alternately almost perpendicular to each other. Both metal atoms display tetrahedron coordination in a selenium-rich environment, comparatively, the coordination geometry of the silver atoms (Se—Ag—Se: 97.67 (5)—115.91 (3)°) is more distorted than the tungsten atoms (Se—W—Se: 106.25 (3)—115.47 (5)°). The chain has a straight linear configuration with an Ag—W—Ag angle of 180°. The average W—µ-Se and Ag—µ-Se bond lengths are 2.3363 (9) and 2.6217 (10) Å, respectively. The average W···Ag distance of 2.9725 (11) Å in the title heteroselenometallic complex is comparable to those in {[Et_4_N][(µ-WSe_4_)Ag]}_n_ (av. 3.0169 (2) Å) (Dai *et al.*, 2007), {[La(Me_2_SO)_8_][(µ_3_-WSe_4_)_3_Ag_3_]}_n_ (av. 3.0038 (12) Å) (Zhang, *et al.*, 2000), and [(µ-WSe_4_)(AgPPh_3_){Ag(PPh_3_)_2_}] (av. 2.996 (1) Å) (Zhang *et al.*, 2002). The hydrogen-bonding interactions exist between the nitrogen atom of pyridinium caion and the nitrogen atom of the pyridine molecule, forming a molecular [(2-MepyH)(2-Mepy)]^+^ cation with the N—H···N distance and angle of 2.786 (12) Å and 171.9 (3)°, respectively. Relatively weak interactions exist between organic cations and polymeric anions *via* the C—H···Se hydrogen-bonds with the C—H···Se distance and angle of 3.731 (2) Å and 141.9 (3)°, respectively, as shown in Fig. 1.

### 2. Experimental

A solution of AgNO_3_ (42.5 mg, 0.25 mmol) in MeCN (5 ml) was added dropwise to a solution of [Et_4_N]_2_[WSe_4_] (190 mg, 0.25 mmol) in DMF (5 ml). The mixture was stirred for 30 min at room temperature, resulting in a large amount of black precipitate. Upon addition of 2 ml 2-picoline solution, the black precipitate was re-dissolved. The resultant solution was stirred for additional 30 min at room temperature and filtered to afford a dark red filtrate. Dark-red prism crystals of the title complex were obtained after allowing the filtrate to stand at room temperature for three days. Anal. Calc. for C_12_H_15_N_2_Se_4_WAg: C, 18.13; H, 1.90; N, 3.53%. Found: C, 18.05; H, 1.87; N, 3.49%.

### 3. Refinement

H atoms were placed in geometrically idealized positions and refined in a riding model with N—H = 0.86, C—H = 0.93–0.97 Å, *U*_iso_(H) = 1.5*U*_eq_(C) for methyl H atoms and 1.2*U*_eq_(C,N) for the others. N-bound H atom has 0.5 site occupancy in the crystal.

### Crystal data

**Table:**

(C_6_H_8_N)[AgWSe_4_]·C_6_H_7_N	*F*(000) = 716
*M**_r_* = 794.82	*D*_x_ = 2.902 Mg m^−^^3^
Monoclinic, *P*2/*c*	Mo *K*α radiation, λ = 0.71073 Å
Hall symbol: -P 2yc	Cell parameters from 1243 reflections
*a* = 7.859 (2) Å	θ = 2.6–24.5°
*b* = 5.9448 (15) Å	µ = 15.39 mm^−^^1^
*c* = 19.830 (5) Å	*T* = 296 K
β = 100.962 (3)°	Prism, dark red
*V* = 909.5 (4) Å^3^	0.15 × 0.12 × 0.03 mm
*Z* = 2	

### Data collection

**Table:**

Bruker APEXII CCD area-detector diffractometer	2051 independent reflections
Radiation source: fine-focus sealed tube	1565 reflections with *I* > 2σ(*I*)
Graphite monochromator	*R*_int_ = 0.041
phi and ω scans	θ_max_ = 27.4°, θ_min_ = 2.6°
Absorption correction: multi-scan (*SADABS*; Bruker, 2001)	*h* = −10→6
*T*_min_ = 0.206, *T*_max_ = 0.655	*k* = −7→7
5398 measured reflections	*l* = −24→25

### Refinement

**Table:**

Refinement on *F*^2^	Primary atom site location: structure-invariant direct methods
Least-squares matrix: full	Secondary atom site location: difference Fourier map
*R*[*F*^2^ > 2σ(*F*^2^)] = 0.034	Hydrogen site location: inferred from neighbouring sites
*wR*(*F*^2^) = 0.078	H-atom parameters constrained
*S* = 0.97	*w* = 1/[σ^2^(*F*_o_^2^) + (0.0357*P*)^2^] where *P* = (*F*_o_^2^ + 2*F*_c_^2^)/3
2051 reflections	(Δ/σ)_max_ = 0.001
93 parameters	Δρ_max_ = 0.86 e Å^−^^3^
0 restraints	Δρ_min_ = −1.06 e Å^−^^3^

### Special details

**Table:**

Geometry. All e.s.d.'s (except the e.s.d. in the dihedral angle between two l.s. planes) are estimated using the full covariance matrix. The cell e.s.d.'s are taken into account individually in the estimation of e.s.d.'s in distances, angles and torsion angles; correlations between e.s.d.'s in cell parameters are only used when they are defined by crystal symmetry. An approximate (isotropic) treatment of cell e.s.d.'s is used for estimating e.s.d.'s involving l.s. planes.
Refinement. Refinement of *F*^2^ against ALL reflections. The weighted *R*-factor *wR* and goodness of fit *S* are based on *F*^2^, conventional *R*-factors *R* are based on *F*, with *F* set to zero for negative *F*^2^. The threshold expression of *F*^2^ > σ(*F*^2^) is used only for calculating *R*-factors(gt) *etc*. and is not relevant to the choice of reflections for refinement. *R*-factors based on *F*^2^ are statistically about twice as large as those based on *F*, and *R*- factors based on ALL data will be even larger.

### Fractional atomic coordinates and isotropic or equivalent isotropic displacement parameters (Å^2^)

**Table:**

	*x*	*y*	*z*	*U*_iso_*/*U*_eq_	Occ. (<1)
W1	0.5000	0.30930 (6)	0.7500	0.03027 (13)	
Ag1	0.5000	0.80929 (13)	0.7500	0.0524 (2)	
Se1	0.24423 (10)	0.09964 (13)	0.72794 (4)	0.0417 (2)	
Se2	0.50518 (11)	0.51956 (12)	0.65078 (4)	0.0434 (2)	
N1	0.0998 (7)	0.3192 (10)	0.4854 (3)	0.0389 (14)	
H1N	0.0423	0.4382	0.4914	0.047*	0.50
C1	0.1778 (10)	0.1984 (14)	0.5389 (4)	0.0478 (19)	
H1	0.1711	0.2486	0.5828	0.057*	
C2	0.2676 (11)	0.0041 (13)	0.5329 (5)	0.053 (2)	
H2	0.3163	−0.0786	0.5716	0.064*	
C3	0.2838 (12)	−0.0653 (16)	0.4690 (5)	0.062 (2)	
H3	0.3473	−0.1938	0.4635	0.075*	
C4	0.2043 (11)	0.0586 (15)	0.4116 (4)	0.053 (2)	
H4	0.2133	0.0114	0.3677	0.064*	
C5	0.1125 (10)	0.2507 (14)	0.4203 (4)	0.0447 (19)	
C6	0.0282 (12)	0.3915 (15)	0.3622 (4)	0.059 (2)	
H6A	0.0500	0.3291	0.3200	0.088*	
H6B	0.0741	0.5414	0.3678	0.088*	
H6C	−0.0945	0.3955	0.3610	0.088*	

### Atomic displacement parameters (Å^2^)

**Table:**

	*U*^11^	*U*^22^	*U*^33^	*U*^12^	*U*^13^	*U*^23^
W1	0.0371 (2)	0.0225 (2)	0.0303 (2)	0.000	0.00409 (16)	0.000
Ag1	0.0704 (6)	0.0280 (4)	0.0565 (5)	0.000	0.0061 (5)	0.000
Se1	0.0412 (4)	0.0389 (4)	0.0425 (4)	−0.0057 (3)	0.0020 (3)	0.0049 (3)
Se2	0.0587 (5)	0.0342 (4)	0.0377 (4)	−0.0027 (4)	0.0101 (4)	0.0051 (3)
N1	0.035 (3)	0.046 (4)	0.035 (3)	−0.005 (3)	0.004 (3)	−0.005 (3)
C1	0.049 (5)	0.060 (5)	0.034 (4)	−0.001 (4)	0.005 (4)	−0.005 (4)
C2	0.056 (5)	0.039 (5)	0.064 (5)	0.005 (4)	0.010 (5)	0.010 (4)
C3	0.053 (5)	0.053 (6)	0.089 (7)	0.004 (5)	0.032 (5)	−0.007 (5)
C4	0.052 (5)	0.059 (6)	0.051 (5)	−0.009 (5)	0.016 (4)	−0.015 (4)
C5	0.040 (4)	0.054 (5)	0.040 (4)	−0.008 (4)	0.007 (3)	−0.004 (4)
C6	0.062 (6)	0.078 (6)	0.033 (4)	−0.006 (5)	−0.001 (4)	−0.008 (4)

### Geometric parameters (Å, º)

**Table:**

W1—Se1	2.3347 (9)	N1—H1N	0.8600
W1—Se1^i^	2.3347 (9)	C1—C2	1.371 (10)
W1—Se2^i^	2.3379 (9)	C1—H1	0.9300
W1—Se2	2.3379 (9)	C2—C3	1.361 (12)
W1—Ag1	2.9723 (11)	C2—H2	0.9300
W1—Ag1^ii^	2.9725 (11)	C3—C4	1.400 (12)
Ag1—Se1^iii^	2.6224 (11)	C3—H3	0.9300
Ag1—Se1^iv^	2.6224 (11)	C4—C5	1.380 (11)
Ag1—Se2	2.6210 (10)	C4—H4	0.9300
Ag1—Se2^i^	2.6210 (10)	C5—C6	1.475 (11)
Ag1—W1^iv^	2.9725 (11)	C6—H6A	0.9600
N1—C1	1.330 (9)	C6—H6B	0.9600
N1—C5	1.376 (9)	C6—H6C	0.9600
			
Se1—W1—Se1^i^	115.47 (5)	Se1^iv^—Ag1—W1^iv^	48.84 (2)
Se1—W1—Se2^i^	106.92 (3)	W1—Ag1—W1^iv^	180.0
Se1^i^—W1—Se2^i^	106.25 (3)	W1—Se1—Ag1^ii^	73.43 (3)
Se1—W1—Se2	106.25 (3)	W1—Se2—Ag1	73.40 (3)
Se1^i^—W1—Se2	106.92 (3)	C1—N1—C5	119.0 (6)
Se2^i^—W1—Se2	115.36 (4)	C1—N1—H1N	120.5
Se1—W1—Ag1	122.27 (2)	C5—N1—H1N	120.5
Se1^i^—W1—Ag1	122.27 (2)	N1—C1—C2	123.5 (7)
Se2^i^—W1—Ag1	57.68 (2)	N1—C1—H1	118.2
Se2—W1—Ag1	57.68 (2)	C2—C1—H1	118.2
Se1—W1—Ag1^ii^	57.73 (2)	C3—C2—C1	118.5 (8)
Se1^i^—W1—Ag1^ii^	57.73 (2)	C3—C2—H2	120.7
Se2^i^—W1—Ag1^ii^	122.32 (2)	C1—C2—H2	120.7
Se2—W1—Ag1^ii^	122.32 (2)	C2—C3—C4	119.4 (8)
Ag1—W1—Ag1^ii^	180.000 (1)	C2—C3—H3	120.3
Se2—Ag1—Se2^i^	97.84 (4)	C4—C3—H3	120.3
Se2—Ag1—Se1^iii^	115.91 (3)	C5—C4—C3	119.8 (7)
Se2^i^—Ag1—Se1^iii^	115.35 (3)	C5—C4—H4	120.1
Se2—Ag1—Se1^iv^	115.35 (3)	C3—C4—H4	120.1
Se2^i^—Ag1—Se1^iv^	115.91 (3)	N1—C5—C4	119.7 (7)
Se1^iii^—Ag1—Se1^iv^	97.67 (5)	N1—C5—C6	117.6 (7)
Se2—Ag1—W1	48.92 (2)	C4—C5—C6	122.7 (7)
Se2^i^—Ag1—W1	48.92 (2)	C5—C6—H6A	109.5
Se1^iii^—Ag1—W1	131.16 (2)	C5—C6—H6B	109.5
Se1^iv^—Ag1—W1	131.16 (2)	H6A—C6—H6B	109.5
Se2—Ag1—W1^iv^	131.08 (2)	C5—C6—H6C	109.5
Se2^i^—Ag1—W1^iv^	131.08 (2)	H6A—C6—H6C	109.5
Se1^iii^—Ag1—W1^iv^	48.84 (2)	H6B—C6—H6C	109.5

Symmetry codes: (i) −*x*+1, *y*, −*z*+3/2; (ii) *x*, *y*−1, *z*; (iii) −*x*+1, *y*+1, −*z*+3/2; (iv) *x*, *y*+1, *z*.

### Hydrogen-bond geometry (Å, º)

**Table:**

*D*—H···*A*	*D*—H	H···*A*	*D*···*A*	*D*—H···*A*
N1—H1*N*···N1^v^	0.86	1.93	2.786 (12)	172
C1—H1···Se1	0.93	2.96	3.732 (8)	141
C4—H4···Se1^vi^	0.93	2.90	3.832 (8)	176

Symmetry codes: (v) −*x*, −*y*+1, −*z*+1; (vi) *x*, −*y*, *z*−1/2.
